# Anesthetic Consideration for Modified Electroconvulsive Therapy in an Adolescent With Uncorrected Atrial Septal Defect: A Case Report

**DOI:** 10.7759/cureus.73753

**Published:** 2024-11-15

**Authors:** Vikash Bansal, Kausiki Poddar, Sanjay Kumar, Rishabh Jaju, Habib Md R Karim

**Affiliations:** 1 Anaesthesiology, Critical Care and Pain Medicine, All India Institute of Medical Sciences, Deoghar, IND; 2 Anaesthesiology, Critical Care and Pain Medicine, All India Institute of Medical Sciences, Guwahati, IND

**Keywords:** atrial septal defect (asd), bfcrs scores, congenital heart diseases, electroconvulsive therapy (ect), etomidate

## Abstract

Catatonia is a serious neuropsychiatric syndrome of motor and behavioral dysfunction where electroconvulsive therapy (ECT) is a well-proven treatment modality. ECT is also preferred as it is a low-risk procedure compared to chronic medications having significant side effects. However, the cardiovascular events that occur during ECT are a major cause of morbidity and mortality in patients with an abnormal cardiovascular pathophysiology. Hence, ECT administration in patients with cardiac diseases remains debatable. Limited literature is present on the use of ECT in a patient with congenital heart disease, especially in uncorrected Atrial Septal Defect (ASD), which is considered a high-risk constellation for an ECT treatment. The goal of anesthetic management in such patients is to maintain pulmonary vascular resistance while slightly reducing the systemic vascular resistance. Currently, modified ECT is performed to reduce the electrical stimulation-related musculoskeletal complications, where the ECT is performed under anesthesia and muscle relaxant. Here we present a case of an 18-year-old male with catatonia with persistent mutism with ASD who underwent six cycles of modified ECT safely. The case highlights the safe use of ECT in uncorrected ASD and the need for a multidisciplinary team.

## Introduction

Electroconvulsive therapy (ECT) is recognized as an effective treatment option for acute schizophrenia (especially with affective/catatonic symptoms), major depressive disorder, bipolar disorder, and schizoaffective disorder. Usually, ECT is reserved for those having severe, life-threatening disorders and is usually resistant to pharmacologic treatment [1]. Although there are no absolute contraindications, major adverse cardiac events following ECT are noted in nearly 1 in 50 patients [2]. The procedure is riskier in cardiac patients [3]. Considering the cardiac complications being frequent, including cardiac arrhythmias, myocardial infarction and arrest, and higher incidences in patients with cardiac pathologies, ECT therapy needs to be performed with utmost vigilance and in a center with a multidisciplinary approach [4]. The birth prevalence of congenital heart disease in India is 9/1000 live births [5].

Further, there is an increasing trend of new cases of neuropsychiatric disorders and deaths over time [6]. Therefore, healthcare providers are likely to encounter a patient with cardiologic comorbidities that might pose a challenge, especially for ECT. With the advancement of healthcare and better accessibility, finding an uncorrected congenital heart disease until adulthood is becoming less prevalent. There is a lack of data regarding the safety of ECT in cardiac patients, especially with uncorrected atrial septal defect (ASD). We present the case intending to highlight the safe use of anesthesia for modified ECT in a patient with catatonia and uncorrected ASD and the need for a multidisciplinary team.

## Case presentation

A young male (18 years old, 50 kg, 165 cm, body mass index- 18.4 kg/m^2^) complained of reduced oral intake, decreased interaction, and irritability over the last four months. He kept repeating the announcement made at the railway station or saying words from another person. He maintained fixed postures and stared constantly. Initial management was attempted in another hospital with some improvement, but he was referred to our institute for further relapse. The Bush-Francis Catatonia Rating Scale (BFCRS) observed a score of 14, confirming the diagnosis of catatonia. Intravenous lorazepam 1mg was administered, which showed transient improvement, so another 2mg was administered slowly, which resulted in improvement in posturing and staring. The patient was then prescribed the tablet Lorazepam 2mg each in the morning and evening and just before sleep. Furthermore, the tablet Olanzapine 10mg was given at night. Although the psychotic and affective symptoms, including posturing, improved to some extent, mutism persisted, and hence, modified ECT was planned.

**Figure 1:**
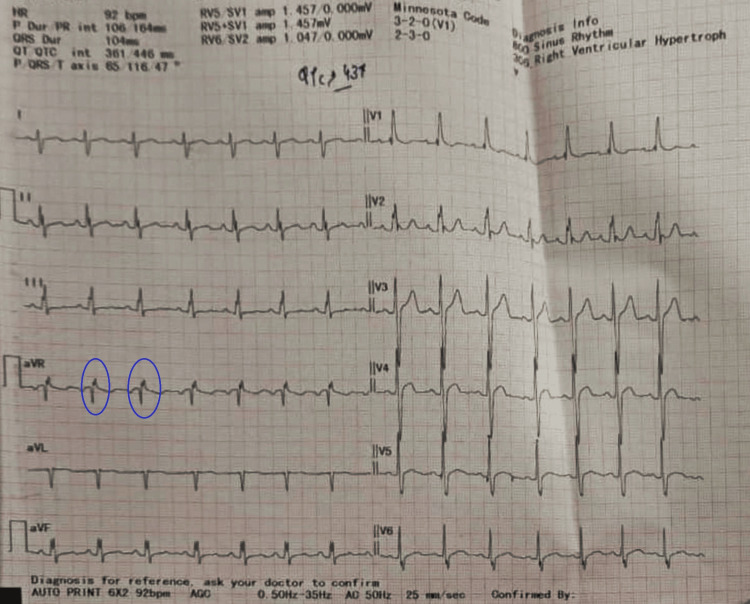
12-lead electrocardiogram showed a right bundle branch block pattern in V1, V2, aVR (pattern is encircled), and a marginally prolonged QTc interval of 446 msec.

On pre-anesthetic evaluation, vitals were within the normal limit, i.e., heart rate of 82/min, blood pressure (BP) 112/63 mmHg, and respiration was normal in rate and pattern. The patient was, however, uncooperative, not making eye contact, and showed mutism, along with a persistent BFCRS score of 14. On cardiorespiratory examination, parasternal heave and ejection systolic murmur of grade-3 was present over the pulmonary valve region, with wide fixed S2. His functional status for cardiopulmonary reserve assessment from clinical assessment was not feasible due to mutism and uncooperative status. Complete blood counts and electrolytes were within normal range, but resting 12-lead electrocardiogram showed a right bundle branch block pattern in V1, V2, aVR, and a marginally prolonged QTc interval of 446 msec (Figure 1).

**Figure 2:**
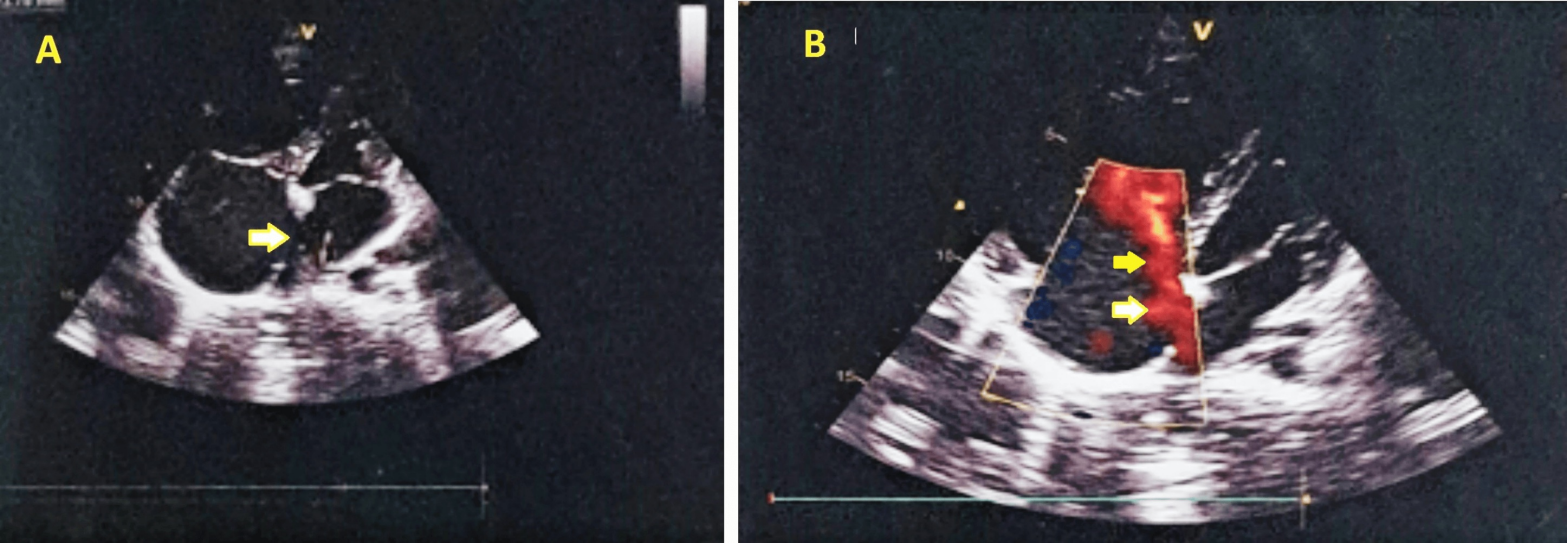
2D Echocardiography showed 25mm Ostium Primum Atrial Septal Defect, dilated Right atrium and right ventricle (A), and mild tricuspid regurgitation (B).

2D echocardiography showed 25 mm ostium primum ASD with left to right shunt, dilated right atrium and right ventricle, mild tricuspid regurgitation (pulmonary artery pressure-35 mmHg), and left ventricular ejection fraction of 55%; no regional wall motion abnormality was detected (Figure 2).

The procedure was performed under the American Society of Anesthesiologists’ standard monitoring recommendation. Anesthesia was induced with etomidate 10mg (IV) and 50 mg of succinylcholine, and ventilation was maintained with a face mask with Bain’s circuit. The left lower limb was isolated using a tourniquet before administering succinylcholine. A generalized tonic/clonic seizure was induced with a stimulus of 60mcCurie, which lasted for 25 sec for the first session. Sevoflurane with 100% O2 was started after the shock to maintain a MACage of 0.3-0.4, which was switched off when motor power returned to normal. The patient showed significant improvement with 6 ECTs, given over two weeks, with a mean seizure duration of 30 seconds; Table 1 shows the summary.

**Table 1:** Showing the summary of the medications, current and seizure duration for different sittings of electroconvulsive therapy.

Session	Etomidate	Succinylcholine	Current	Seizure duration
First (Day 0)	10 mg	50 mg	60 mc	25 sec
Second (Day 2)	10 mg	50 mg	30 mc	23 sec
Third (Day 5)	16 mg	50 mg	60 mc	20 sec
Fourth (Day 7)	10 mg	50 mg	60 mc	24 sec
Fifth (Day 9)	12 mg	50 mg	60 mc	19 sec
Sixth (Day 14)	10 mg	50 mg	60 mc	42 sec

His subsequent BFCRS scores decreased to two. No significant bradycardia or tachycardia was noted during any of the sessions. The highest blood pressure was 130/82 mmHg, and the lowest saturation was 96%. No episode of heart rate below 60/min was noted during the entire sessions of ECTs.

## Discussion

While modified ECT is conducted day in and out, very little literature is known about modified ECT in patients with uncorrected ASD. Our case indicates that the modified ECT can be well conducted without significant hemodynamic instability using etomidate, succinylcholine, and sevoflurane. However, cardiac disease predisposes a patient to major cardiac adverse events during modified ECT, and therefore, thorough knowledge about the changes in cardiac physiology that occur during ECT is required for a better understanding of the detrimental consequences [7]. First, a parasympathetic discharge occurs within a few seconds of the application of the electrical stimulus. This may result in bradycardia, which may even progress to asystole. Because many patients are volume-depleted due to poor appetite and oral intake, they can be susceptible to further hypotension due to anesthetic induction. Within 1 minute, sympathetic activation due to the seizure results in tachycardia and sometimes dysrhythmias and hypertension up to 150% of basal values. This hypertension usually lasts between 2 and 5 minutes. If the patient is not adequately ventilated, hypercarbia may add to hypertension [7]. Etomidate is a very cardio-stable intravenous anesthetic agent. We started the sevoflurane immediately and maintained it at 0.3-0.4 MACage after the current delivery so that the sympathetic surge-related hemodynamic deterioration could be reduced to some extent while keeping the patient conscious.

Varied literature was observed regarding the use of ECT. Kufner et al. reported the safety of ECT without severe side effects in a bipolar patient with hypertrophic cardiomyopathy (HCM) [8]. Adabag et al. established the risk of arrhythmia associated with ECT in HCM with heart failure during the catecholamine surge phase [9]. Ali and Tidmarsh reported cardiac rupture in a 57-year-old man with recurrent depression after a course of ECTs [10]. Grover et al. in 2012 reported a successful ECT in an individual with 25 mm ostium secundum type ASD with significant left-to-right shunt, severe tricuspid regurgitation, and moderate pulmonary artery hypertension. The authors used Glycopyrrolate (0.2 mg) as a premedication, thiopental sodium for induction, and succinylcholine was used for muscle relaxation. The patient received 7 ECT sessions without any cardiac or any other complication, and the patient showed gradual improvement in psychotic and affective symptoms from the second ECT onward [11]. Mohan and Sundram also reported ECT therapy in a patient with a massive ostium secundum ASD, a left to right shunt, a substantially dilated right ventricle, mild right ventricular systolic dysfunction, and a severely dilated pulmonary artery. The therapy did not exacerbate her cardiac or other health issues, and her psychotic and affective symptoms also rapidly improved. Eight bi-temporal ECT sessions were administered four months later [12]. Our patient also had pulmonary hypertension, but the ASD was ostium primum type and uncorrected. Kovvuri et al. successfully conducted ECT in a patient with ventricular septal defect patch using thiopental and succinylcholine [7]. Ikpot et al. successfully performed ECT in a patient after Fontan repair having pulmonary stenosis, tricuspid hypoplasia with a hypoplastic right ventricle, and intact ventricular septum using etomidate as an induction agent [13].

In patients with ASDs, a mild decrease in systemic vascular resistance (SVR) with maintained pulmonary vascular resistance is desirable. Still, a precipitous fall in SVR should be avoided because of potential hemodynamic instability. Moreover, increased SVR can also affect the shunt fraction, which is undesirable [14]. Etomidate maintains a stable SVR instead of the usual regime of using propofol as an intravenous induction agent [13, 15]. ASD patients usually present with a left-to-right shunt, but occasionally, a right-to-left shunt may cause air to enter the systemic circulation and result in a paradoxical cerebral air embolism. Therefore, the goal of anesthetic management in such patients is to lower pulmonary vascular resistance (PVR) as well.

Further, utmost care is required while injecting any drugs to void air entrainment. To prevent an increase in the PVR, acidosis should be prevented or corrected, hypoventilation and sympathetic nervous system stimulation should be avoided, and normothermia should be maintained. Although monitoring like end-tidal carbon dioxide (EtCO2), and peak inspiratory pressure is crucial for PVR and SVR management, modified ECT mostly does not require definitive airway management making these monitoring usually unreliable. Nevertheless, the duration of paralysis and even anesthesia is very short to cause any major derangement of EtCO2. Notably, patient-related factors like age and cardiovascular functional status might affect how the heart responds to ECT, as Chang et al. showed recently using real-time visualization of cardiac function during ECT using point-of-care ultrasound [16]. As major cardiac events, including myocardial infarction and arrest, remain a possibility, although remote, such modified ECT for such patients is better performed in a center with cardiology backup. A multidisciplinary approach, which includes anaesthesiologists, psychiatrists, and cardiologists, should be there in pre-, intra-, and post-operative periods.

## Conclusions

This case report suggests that ECT can be delivered in patients with uncorrected ASD with mild pulmonary hypertension with an effective and multidisciplinary approach. Etomidate provides stable hemodynamics, and adding low-dose sevoflurane to the modified ECT might provide the advantage of dose-dependant SVR reduction. A review of other cases of ECTs in patients with ASD and the current case indicate that modified ECTs using currently used intravenous and volatile anesthetic drugs and monitoring can safely be done. The absence of any hypertension, hypotension, or arrhythmias during the six sessions of modified ECT with etomidate, sevoflurane, and succinylcholine hints towards the safety of the drug combinations. However, it needs to be used further in such congenital heart disease patients before we can firmly claim it.
